# High performance multifunction-in-one optoelectronic device by integrating graphene/MoS_2_ heterostructures on side-polished fiber

**DOI:** 10.1515/nanoph-2021-0688

**Published:** 2022-02-21

**Authors:** Linqing Zhuo, Dongquan Li, Weidong Chen, Yu Zhang, Wang Zhang, Ziqi Lin, Huadan Zheng, Wenguo Zhu, Yongchun Zhong, Jieyuan Tang, Guoguang Lu, Wenxiao Fang, Jianhui Yu, Zhe Chen

**Affiliations:** Guangdong Provincial Key Laboratory of Optical Fiber Sensing and Communications, Department of Optoelectronic Engineering, Jinan University, Guangzhou 510632, China; Guangdong Vocational College of Posts and Telecom, Guangzhou 510630, China; Key Laboratory of Optoelectronic Information and Sensing Technologies of Guangdong Higher Education Institutes, Department of Optoelectronic Engineering, Jinan University, Guangzhou 510632, China; Science and Technology on Reliability Physics and Application of Electronic Component Laboratory, China Electronic Product Reliability and Environmental Testing Research Institute, Guangzhou 510610, China

**Keywords:** graphene/MoS_2_ heterostructures, multifunctional device, photodetector, polarizer, side-polished fiber (SPF)

## Abstract

Two-dimensional (2D) materials exhibit fascinating and outstanding optoelectronic properties, laying the foundation for the development of novel optoelectronic devices. However, ultra-weak light absorption of 2D materials limits the performance of the optoelectronic devices. Here, a structure of MoS_2_/graphene/Au integrated onto the side-polished fiber (SPF) is proposed to achieve a high-performance fiber-integrated multifunction-in-one optoelectronic device. It is found that the device can absorb the transverse magnetic (TM) mode guided in the SPF and generate photocurrents as a polarization-sensitive photodetector, while the transverse electric (TE) mode passes with low loss through the device, making the device simultaneously a polarizer. In the device, the MoS_2_ film and the Au finger electrode can enhance the TM absorption by 1.75 times and 24.8 times, respectively, thus allowing to achieve high performance: a high photoresponsivity of 2.2 × 10^5^ A/W at 1550 nm; the external quantum efficiency (EQE) of 1.76 × 10^7^%; a high photocurrent polarization ratio of 0.686 and a polarization efficiency of 3.9 dB/mm at C-band. The integration of 2D materials on SPF paves the way to enhance the light–2D material interaction and achieve high performance multifunction-in-one fiber-integrated optoelectronic devices.

## 1 Introduction

Two-dimensional (2D) materials with the properties of flexibility, configurability, and versatility have been successfully applied in some outstanding optoelectronic devices, such as photodetectors, modulators, mode-locked fiber lasers, and pulse-shaping devices [1], [2], [3], [4], [5], [6]. However, the ultra-weak light–matter interaction of monolayer 2D materials limits the performance of the device, leading to low photoresponsivity (<32 A/W) [7], [8], [9] for the monolayer graphene photodetectors. One of the strategies to enhance the light–matter interaction is to construct a heterojunction. Stacking different 2D materials to form van der Walls heterojunctions can provide a way to combine the advantages of different materials for high-performance devices [10, 11]. 2D heterostructures can not only strongly enhance the light absorption, but also can efficiently separate the photo-generated carrier to shorten the response time [12]. Heterostructures composed of transition metal dichalcogenides (TMDs) have attracted significant attention, especially molybdenum disulfide (MoS_2_), which exhibits unique physical properties such as bandgap variation with the number of layers [2, 13], high Seebeck coefficient [14], high photoconductivity [15], and high mechanical strength [16]. Furthermore, monolayer MoS_2_ shows three times stronger two photon absorption coefficient (7.6 × 10^−8^ m/W) than other semiconductors, which can significantly enhance light absorption [2]. MoS_2_ based photodetectors usually possess a high responsivity (at the level of kA·W^−1^ under visible light), but suffer from long response time (∼9 s) due to the low electron mobility (44 cm^2^ V^−1^ s^−1^) [17], [18], [19]. Thus, as shown below, stacked graphene/MoS_2_ heterostructure is an effective way to improve the performance of photodetectors. Another strategy is the waveguide integration, providing an efficient way to enhance the absorption of 2D materials limited by the atom-scale thickness [20, 21]. Using side-polished fiber (SPF) integration, Zhuo et al. has successfully demonstrated an ultra-high responsivity of graphene/PMMA photodetector up to 1.5 × 10^7^ A/W [20], which confirms the SPF can be a versatile and outstanding platform on which the 2D-material could be integrated. Other mechanisms were exploited to enhance the responsivity of graphene photodetectors by using quantum dots modify graphene [22], surface plasmons [23], and coherent control [24]. Similar efforts have been explored on anisotropic 2D-material-based nanoscale polarizers and SPF-integrated polarizers to enhance the light–matter interaction [25, 26]. The first nanoscale polarizer based on anisotropic of the black phosphorus using the Fabry–Perot cavities method is demonstrated to achieve an extinction ratio of ∼9 dB in visible range [25]. Bao et al. integrated graphene onto SPF to design a broadband polarizer with a high extinction ratio of ∼23.6 dB at 1550 nm [26].

Different materials and structures were demonstrated to integrate onto the optical fiber to realize an optical fiber system that integrates seeing, hearing, sensing and communication [27], [28], [29]. Recently, there is booming research interest in the development of multi-functional integration optical fiber devices [20, 21, 30, 31]. By now, some multi-functional optoelectronic devices integrated on optical fiber end-face and SPFs have been reported [20, 21, 31]. Different from achieving multi-functional integration through multiple structures on different optical fiber end-face [31], a photodetector and an optic-phase modulator were shown to be integrated in-one device on an SPF [20]. Dong et al. realized a photodetector and an intensity modulator in-one SPF-based device [21]. To the best of our knowledge, a multifunction-in-one device as a polarization sensitive photodetector and polarizer has not been reported.

Manipulating the polarization of electromagnetic waves is of great significance to fiber optic gyroscope systems, polarization-maintaining fiber amplifiers, machine vision, coherent optical communication systems and information security [32], [33], [34]. To prevent light signal fading, a polarizer has become a key optical passive component in photonic integrated circuits and optical fiber systems [35], [36], [37]. The polarizer usually absorbs an undesired polarization while passes other perpendicular polarization mode [38]. For the usual polarizer, at least half of the light energy will be lost, leading to the large energy consumption in the optical system. On the other hand, polarization sensitive photodetectors are useful in the fields of high-performance optical signal capture and stray light shielding, which can improve the detection and recognition capabilities of targets in complex environments [39, 40]. Conventional photodetectors usually use filters and polarizers to achieve polarization sensitive detection. To simplify the optical system, integration of both polarization-sensitive detection and polarizer into one-device has great significance for high-performance optical signal capture and polarization division multiplexing (PDM) systems [41].

**Figure 1:**
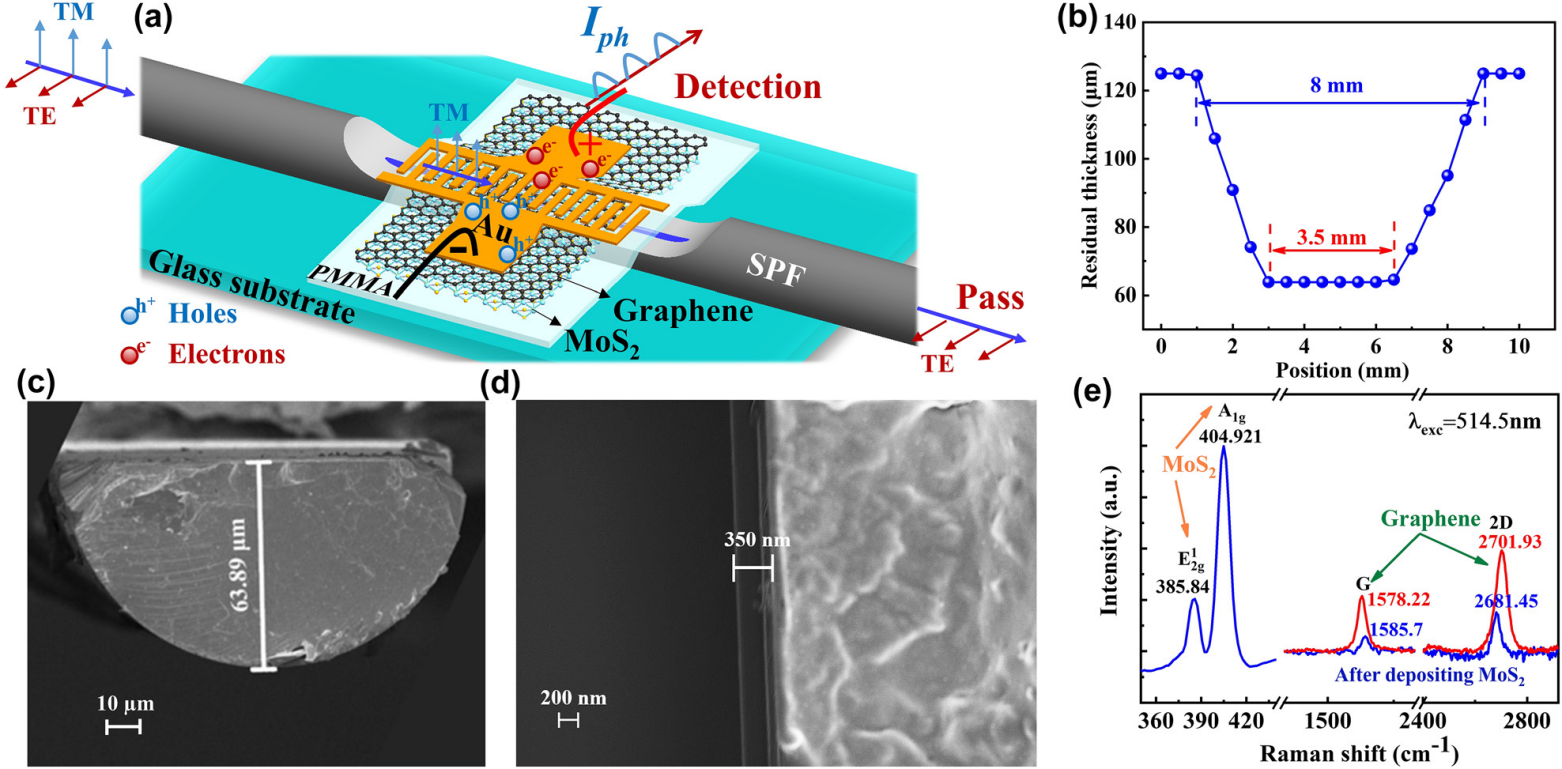
Schematic and characterization. (a) Schematic structure of PPID; (b) morphological characteristic of the SPF along the fiber; (c) SEM image of the cross section of PPID; (d) SEM image at the interface between the graphene/MoS_2_/PMMA film and glass substrate; (e) Raman spectra of the pristine graphene (red curve) and the graphene/MoS_2_ heterostructures (blue curve).

Here, we successfully demonstrated a high-performance fiber-integrated multifunction-in-one device by integrating the graphene/MoS_2_ and Au interdigital electrode onto an SPF. The device can work simultaneously as a polarizer and a polarization-sensitive photodetector. Here, we call it a polarizer/photodetector-in-one device (PPID). The polarizer and polarization-sensitive photodetector functions originated from the much higher absorption of the transverse magnetic (TM) mode than the absorption of the transverse electric (TE) mode caused by the Au film, MoS_2_ film and D-shape structure of the SPF, as shown in Figure 1a. Due to the on-fiber integration, the PPID is capable of seamless connecting with the current mature optical fiber system in a low loss. It is found experimentally that when working as a polarizer, the PPID exhibits an extinction efficiency of 3.9 dB/mm at C-band and a tunable polarization extinction ratio (PER) from 13.7 to 19.2 dB with the bias voltage increase from 0 to 4 V. When working as a photodetector, it shows a broadband photosensitive wavelength from 980 to 1620 nm, and a high photoresponsivity of up to 2.2 × 10^5^ A W^−1^ for a wavelength of 1550 nm at 0.3 V bias. This work paves a way to enhance the ligh–2D-material interaction and achieve high-performance all-fiber multifunction-in-one optoelectronic device through 2D-material on-fiber integration. In addition, the on-fiber integration allows us to avoid the high coupling loss between optical fibers and on-chip integrated devices.

## 2 Results and discussion

The schematic diagram of PPID, working simultaneously as a multi-function of polarization sensitive photodetector and polarizer, is shown in Figure 1a. For a conventional polarizer, a polarization mode is absorbed and other perpendicular polarization mode passes through in very low loss, thus realizing the polarization function. However, the absorbed part of the energy is usually useless and wasted. To overcome the waste issue, we propose a PPID here, where the absorbed photon energy of TM mode is used to generate electron–hole carrier pairs and the photo-carriers are separated under a bias voltage to cause a photocurrent for the photodetection, as shown in Figure 1a. In this work, the SPF is made from a commercial single-mode fiber (SMF-28e, Corning, with its diameter of 125 μm and the diameter of fiber core of 8 μm) by wheel polishing technique. The PPID is fabricated by wet transferring a graphene/MoS_2_/PMMA hybrid film onto the flat polished region of SPF, and then use a mask to form periodically arranged interdigitated Au electrodes on the top of graphene layer by physical vapor deposition. The spacing and finger width of the interdigitated Au finger electrode are 157.4 and 214.6 μm, respectively. The electrodes can be tuned by customizing the spacing and shape of the mask, and the minimum spacing that can be processed is 50 µm. Au electrodes are used to collect the photocurrent and help to enhance the TM mode absorption, and the interdigital structure of the Au electrodes can short the transit time of photo-carriers. The monolayer graphene/MoS_2_ heterostructures (Six Carbon Tech.) is prepared by chemical vapor deposition (CVD) technology. Here, residual thickness is defined as the distance from polished surface of SPF to the bottom of fiber. Figure 1b shows the morphological characteristic of the SPF along the fiber. It can be seen that the residual thickness of this SPF is about 64 μm, and the length of flat and entire polished sections of SPF are 3.5 and 8 mm, respectively, the insertion loss of the bare SPF is 3.2 dB. Figure 1c and d shows the scanning electron microscopic (SEM) image of the cross section of PPID and of the cross section of glass substrate coated graphene/MoS_2_/PMMA film, respectively. Figure 1d shows that the thickness of graphene/MoS_2_/PMMA film on the glass substrate is about 350 nm. Raman spectra (excited at a 514.5 nm laser) of a graphene/MoS_2_ heterostructures is measured with a Raman microscope as shown in Figure 1e. The Raman spectra of pristine graphene are shown in the red curve in Figure 1e. The G (1578.22 cm^−1^) and 2D (2701.93 cm^−1^) peaks in Raman spectra are two typical featured peaks of graphene. After depositing MoS_2_ on graphene to construct graphene/MoS_2_ heterostructures, the G and 2D peak positions downshifted and changed to 1585.7 cm^−1^ and 2681.45 cm^−1^, respectively, it may be caused by strains or charge transfers (shown in the blue curve) [38]. The 2D/G intensity ratio is 2.6 confirms that the graphene layer is monolayer [42]. The Raman peaks, corresponding to parallel direction oscillatory mode E^1^_2g_ and perpendicular direction oscillatory mode A_1g_ of MoS_2_ locate at 385.8 cm^−1^ and 404.9 cm^−1^, respectively. The spectrum difference between E^1^_2g_ and A_1g_ modes is 19.1 cm^−1^, indicating the MoS_2_ is monolayer [39]. The Raman spectrum confirms that a monolayer graphene/MoS_2_ heterostructures film was successfully transferred to the SPF.

**Figure 2:**
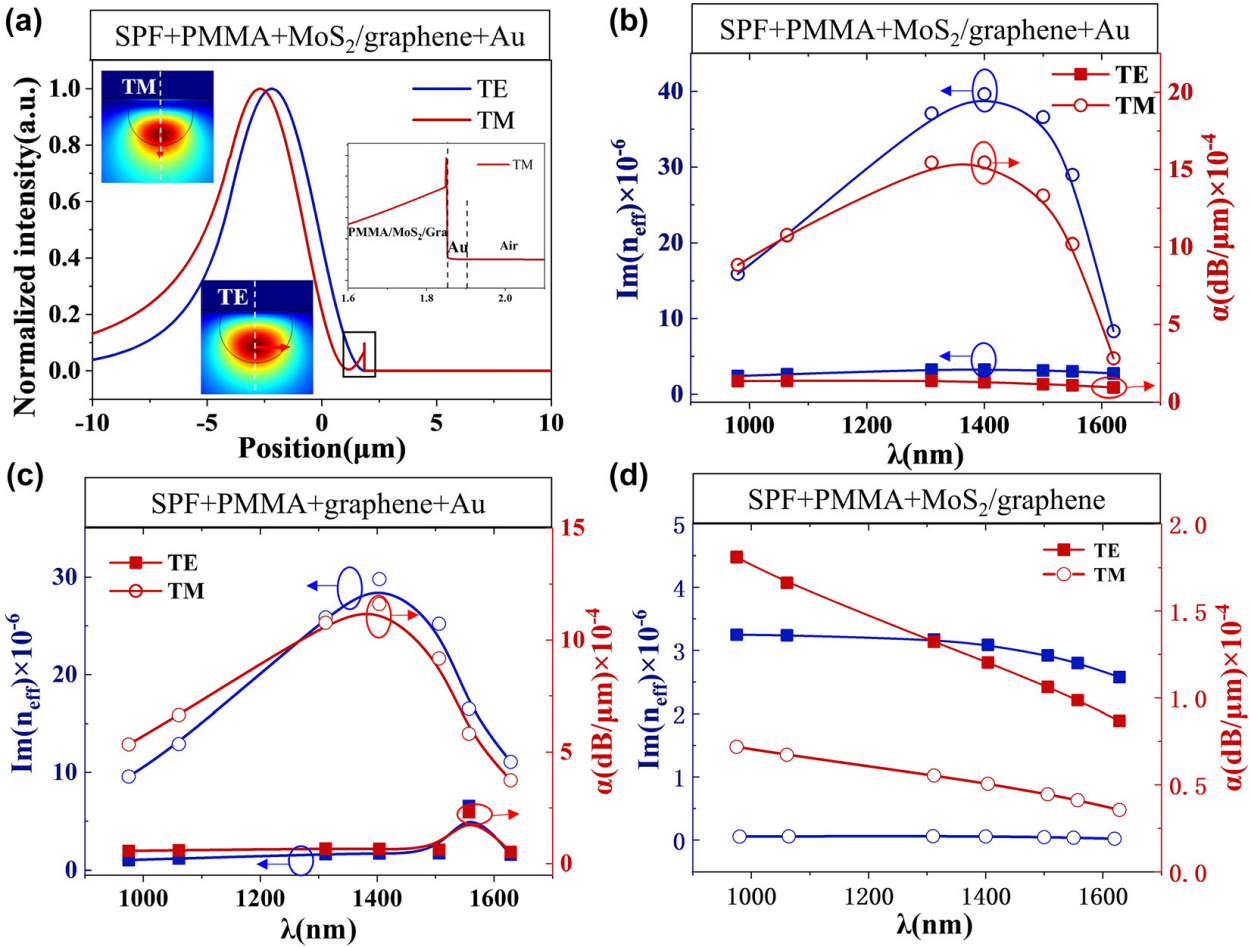
The simulation results. (a) Normalized intensities along the white dotted lines from TM and TE modes, the insets include TM and TE modes optical field distribution at 1550 nm and the enlarge view of TM mode normalized intensity; (b) imaginary parts of the effective refractive indices Im(*n*_eff_) and absorption coefficients *α* as a function of the wavelengths (980, 1064, 1310, 1400, 1500, 1550, and 1620 nm) with MoS_2_/Graphene/Au, (c) without MoS_2_, and (d) without Au film, respectively.

To investigate the enhancement of light–matter interaction by the Au film and MoS_2_ monolayer, we simulated the field mode guided in the PPID by the full vector finite element method (Commercial software Comsol Multiphysics). Figure 2a and b shows the simulation results of Au/graphene/MoS_2_/PMMA/SPF structure by commercial finite element software Comsol Multiphysics. The normalized field intensities along the white dotted lines from TM and TE modes at 1550 nm in the inset are shown in Figure 2a. The right inset is an enlarge view of normalized intensity of TM mode in the location of graphene. The D-shape structure of the SPF leads to different absorption of orthogonal polarizations. According to the simulation results, the normalized intensity distribution of TM mode is 65 times larger than the TE mode at the graphene. This great different light absorption of TM mode and TE mode can be enhanced by Au film and MoS_2_ film [43]. Figure 2c and d shows the detailed contrast the imaginary parts of the effective refractive indices Im(*n*_eff_) and absorption coefficients *α* as the function of wavelengths when the structure has no MoS_2_ and Au film, respectively. The absorption of TM mode is always greater than TE mode in the structure with and without MoS_2_ due to the optical field confinement effect of the Au film, as shown in Figure 2b and c. The absorption coefficients *α* of PPID can be calculated by: $\alpha =40\pi \;\text{Im}\left(n_{\text{eff}}\right)/\lambda \;\text{ln}\;10$ (dB/m), where *λ* is the incident wavelength. Thanks to two-photon absorption of the MoS_2_ film and the light field confinement effect of the Au film, the monolayer MoS_2_ film and Au film exhibit the ability to enhance TM mode absorption. The monolayer MoS_2_ enhances the Im(*n*_eff_) and *α* of TM mode by 1.75 times and decreases the Im(*n*_eff_) and *α* of any enhancement to TE mode. The number of layers in graphene/MoS_2_ heterostructures will influence the absorption of TM and TE modes. It is possible to control the polarization effect by controlling the number of layers of heterojunctions. The 3.5 mm long side-polished region ensures efficient light–matter interaction and the MoS_2_ further enhanced the light absorption. The simulation reveals that the polarizing effect and polarization-sensitivity of the photodetector originates from the much higher absorption of the TM than TE modes.

**Figure 3:**
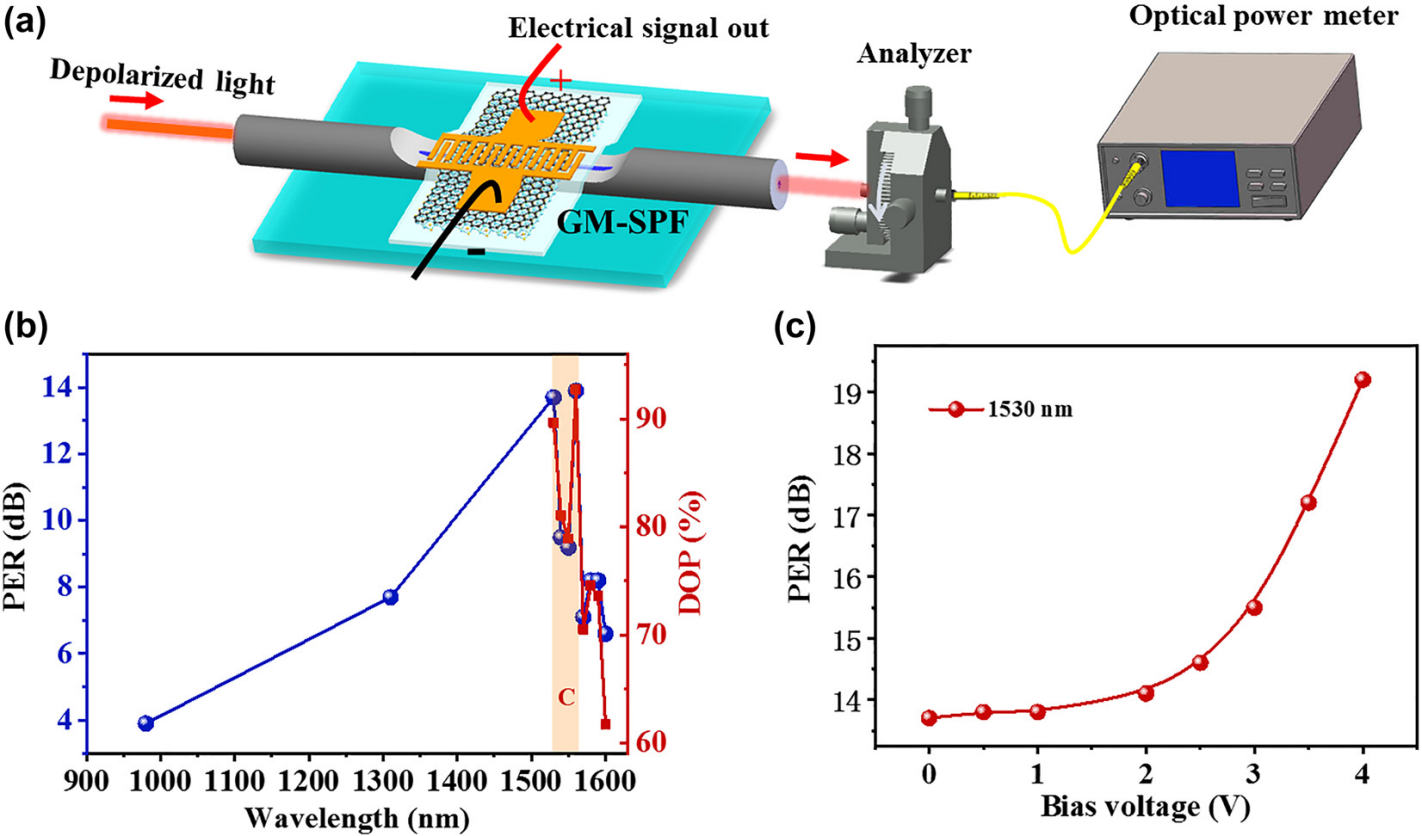
The polarization characteristics of PPID. (a) Schematic diagram of the experimental setup for measuring the polarizing effect of PPID; (b) the PER (blue line) and the DOP (red line) as a function of wavelength at room temperature; (c) the PER increase with the bias voltage from 13.7 to 19.2 dB at 1530 nm.

Figure 3a shows the experimental setup for measuring the polarization characteristics of PPID. The polarization extinction ratio (PER) is measured by a 360° rotating analyzer and an optical power meter. It is expressed as: PER = 10 log 10(*P*_max_/*P*_min_), where *P*_max_ and *P*_min_ are the maximum and minimum output power of the natural light passing through the PPID and then through an analyzer, respectively. The degree of polarization (DOP) is measured as follows. We use a tunable laser operating at 1500–1630 nm and a polarization scrambler to obtain the broadband depolarized light. Then, input the depolarized light into the PPID, and the output light is measured by a polarization analyzer (PSGA-101-A). The PER and DOP of PPID at a wavelength of 980–1600 nm at room temperature are shown in Figure 3b. The DOP is larger than 79% in telecommunication C-band (1530–1565 nm). The PPID shows the broadband polarization characteristics from 1400 to 1560 nm over the entire C-band. The PER reaches 13.7 and 9.5 dB at 1530 and 1550 nm, respectively. The wavelength-dependent PER is due to optical wave interference in the cavity formed by Au and PMMA films. Additionally, the PER can be enhanced by the bias voltage from 13.7 to 19.2 dB when the voltage increases from 0 to 4 V at the wavelength of 1530 nm. The PER increases exponentially with the bias voltage, as illustrated in Figure 3c. The origin of the PER increasing with voltage and the mechanism of the tunable PER by the increasing voltage can be explained as following. The increasing voltage results in the temperature rise of the PPID by the ohmic heating, thus leading to the decrease in the band gap (E_g_) of monolayer MoS_2_ film [44] and thus the macroscopic increase in the conductivity *δ* (decrease in the resistance) of the graphene/MoS_2_ film in the PPID. Because the absorption of the TM mode is proportional to the conductivity *δ* [45], the rise of the temperature leads to a higher PER with increasing voltage.

**Figure 4:**
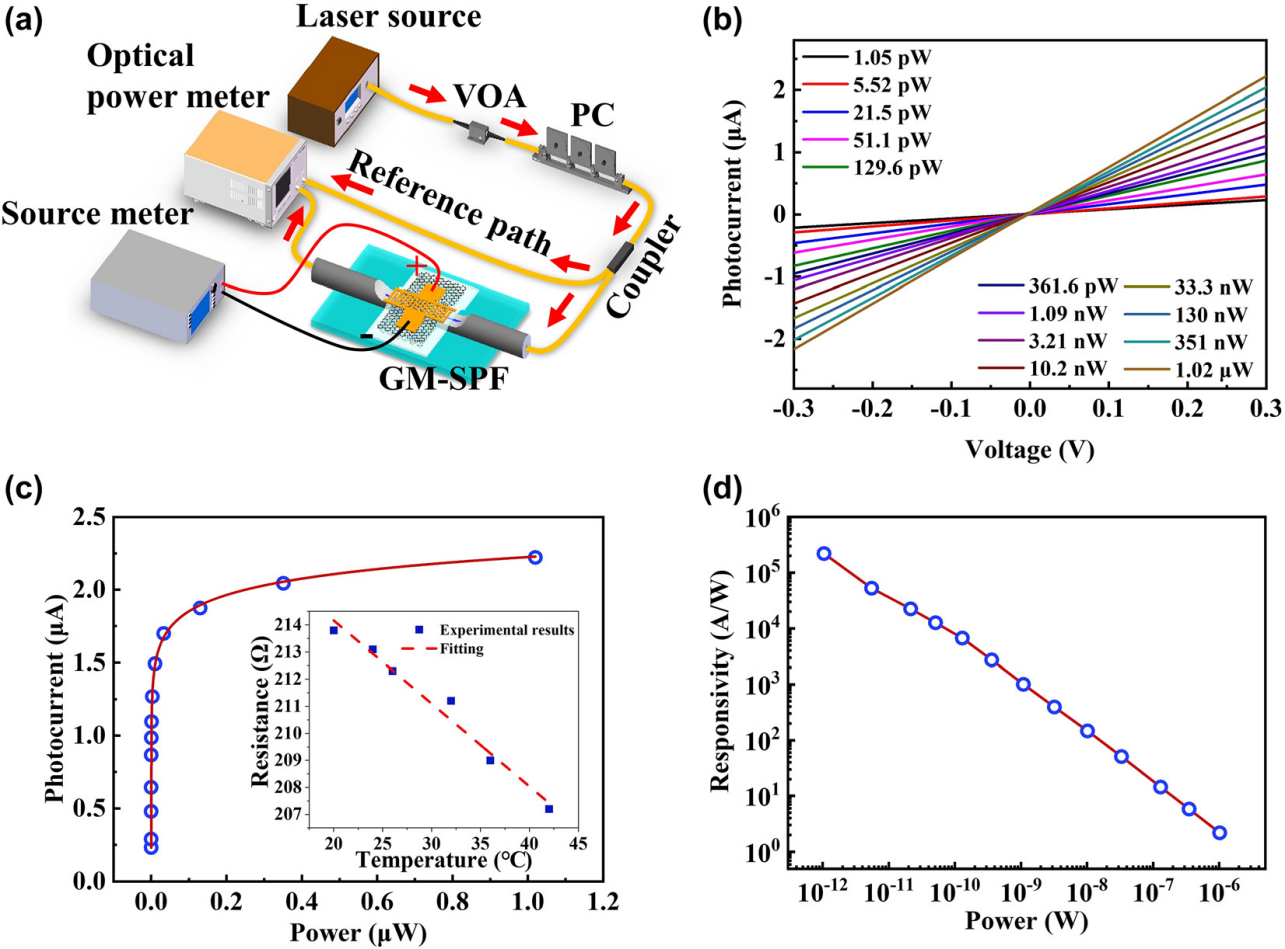
The photodetection characteristics of PPID. (a) Experimental setup of photodetection; (b) relationship between photocurrent and applied voltage of the device for different light powers at a wavelength of 1550 nm; (c) relationship between photocurrent and light power under 0.3 V bias, the inset is the resistance of PPID changing with the rising temperature under 0.3 V; (d) relationship between responsivity and light power under 0.3 V bias.

The schematic of photodetection experimental setup is shown in Figure 4a, a variable fiber-optic attenuator (VOA) is used to control the incident light power, the polarization state is chosen by a polarization controller (PC); the 3 dB coupler separates the light into two arms. One arm connected with a power meter for optical power measurement as reference path; the other is launched into the PPID. The generated electrical signal is collected and analyzed by a source meter. Figure 4b shows the relationship between the photocurrent (*I*_ph_ = *I*_light_ − *I*_dark_) and the bias voltage (*V*_bias_) of PPID with different light powers at 1550 nm. The photocurrents increase linearly with the bias voltage, indicating a good ohmic contact between graphene and Au electrodes.

Here, the photocurrent generation in PPID is thought to be caused by photo-bolometric effect and photoconductive effect [46, 47]. The photocurrent increases rapidly with the low incident power when *V*_bias_ = 0.3 V (photoconductive effect dominates), but when the incident light power increases, the photon absorption of graphene/MoS_2_ reaches saturated due to the Auger processes or the reduced density of available states. Hence, the excess photons will not be absorbed by graphene/MoS_2_ heterojunction beyond the saturated absorption, and the photocurrent will not increase further, as shown in Figure 4c. The temperature of PPID will rise with the increasing incident light power, the resistance of the graphene/MoS_2_ film in the PPID decreases with the rising temperature under a fixed bias voltage of 0.3 V (photo-bolometric effect). Figure 4d shows a linear relationship between the responsivity and incident light power in log–log coordinates. The responsivity of PPID reaches up to 2.2 × 10^5^ A W^−1^ at an incident light power of 1.05 pW with a bias voltage of 0.3 V in room temperature. The ultra-high responsivity benefits from the strong interaction between the light and the MoS_2_/graphene/Au heterostructures due to the optical field confinement effect of the Au film. The external quantum efficiency (EQE) is used to characterize the efficiency of the impinging photons for generating charge carriers. Here the EQE of the PPID reaches 1.76 × 10^7^%. The EQE is represented as the ratio of the number of generating charge carriers and the total number of impinging photons, which is closely related to the responsivity: EQE = *R*(*hc*/*eλ*), where *h* is Planck’s constant, *c* is light speed in a vacuum, *e* is the electron charge, and *λ* is the light wavelength. Here, we package the PPID with inert gas to make sure it can work stability for at least three months.

**Figure 5:**
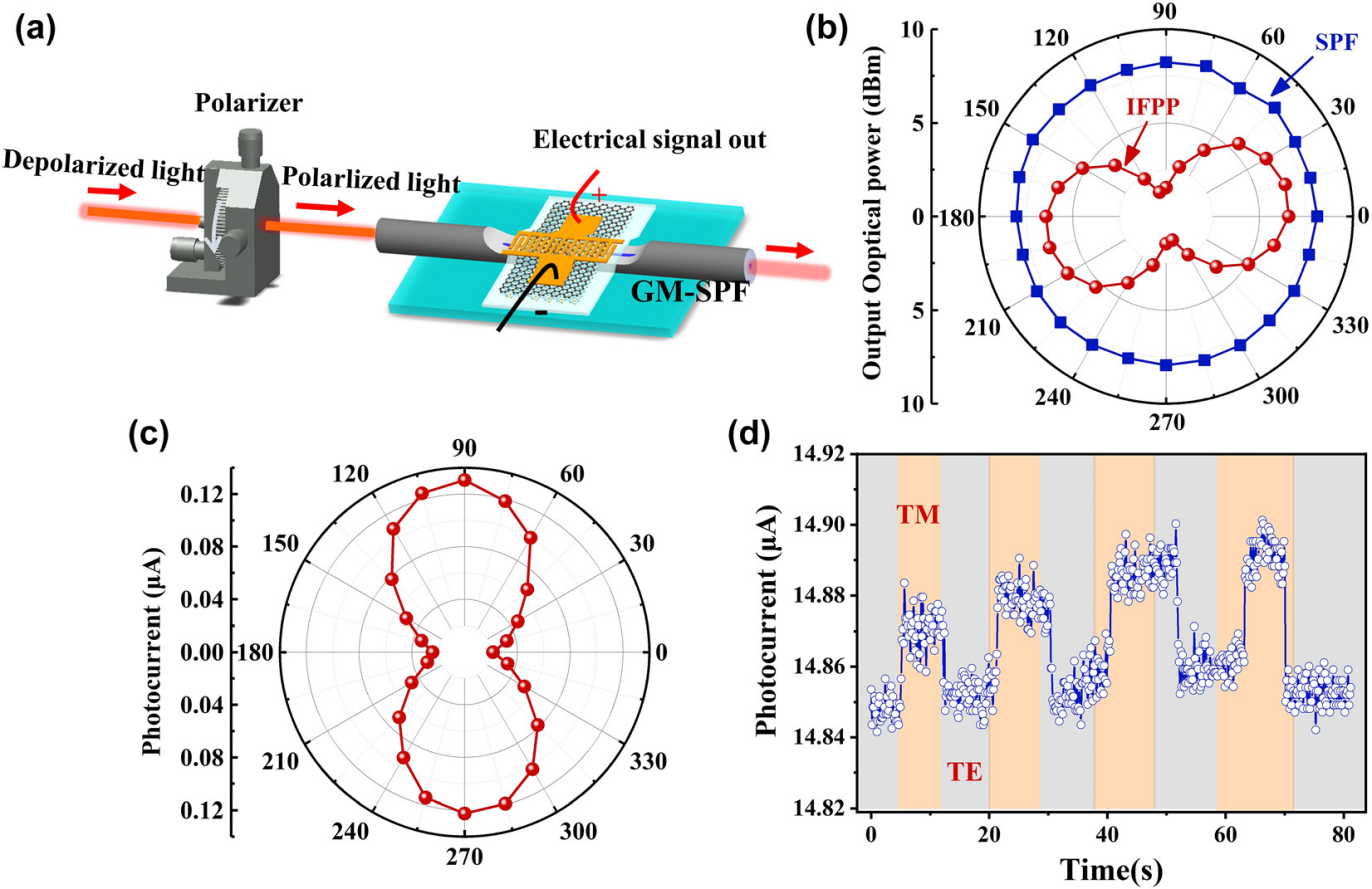
The polarized light detection characteristics of PPID. (a) Schematic diagram of experimental measurement of the polarization sensitivity of the PPID; (b) the output optical power through the PPID (blue squares) and the bare SPF with the different IPA in polar coordinates (red spheres); (c) polarization-dependent property of the photocurrent by the PPID at 1550 nm; (d) the PPID photocurrent when switching repeatedly between TM and TE polarization.

To accurately detect the polarization information on incident light, a 360° rotating polarizer was inserted between the depolarized light and the PPID as shown in Figure 5a. The optical field confinement effect of Au film and asymmetric structure of SPF make PPID highly sensitive to polarized light. Realizing polarization dependence device through this structure is not limited to graphene/MoS_2_ heterostructures, but also applicable to other 2D materials. We measure the output optical power of PPID and a bare SPF, respectively, as shown in Figure 5b. The output optical power of bare SPF remains almost unchanged to exhibit the isotropy behavior. The output optical power of PPID at 90° and 270° incident polarization angles (IPA) were greatly suppressed down to 1.5 ± 0.4 dBm, whereas at 0° and 180° IPA is up to 6.5 ± 0.06 dBm. The total loss, including insertion loss and the effective optical power absorption of PPID, measured at the wavelength of 1550 nm, is 17.58 dB for IPA = 90° or 270°, and 10.5 dB for IPA = 0° or 180°. Accordingly, to rotate the polarizer from 0 to 360°, the photocurrent generated by PPID changes significantly. The photocurrent versus the polarization angle of incident light is shown in polar coordinates; the two-lobed trend indicates the distinct polarization dependence response (Figure 5c). The photocurrent reached maximum values (∼0.1306 µA) at polarization angles of 90° and 270°, and reached minimum values (∼0.0243 µA) at polarization angles of 0° and 180°. The photocurrent values conform to the numerical simulation, the larger absorption of TM mode (90° and 270°) by the PPID caused larger photocurrent. The variation of the photocurrent $\left(I_{\text{max}}-I_{\text{min}}\right)/I_{\text{max}}$ = 0.85, indicating the photocurrent is partly polarized and a high polarization sensitive [48]. Additionally, it also shows that the PPID structure has photocurrent polarization ratio $\gamma =\left(I_{\text{max}}-I_{\text{min}}\right)/\left(I_{\text{max}}+I_{\text{min}}\right)=0.686\text{,}$ which is much larger than the anisotropic absorption from crystal (γ=0.474) [49, 50]. Figure 5d shows the photocurrent generated by the PPID when repeatedly switching polarization states between TM and TE under *V*_bs_ = 0.01 V, *P*_in_ = 2.6 mW. It proves the PPID device can stably and continuously detect two orthogonal polarization states.

**Figure 6:**
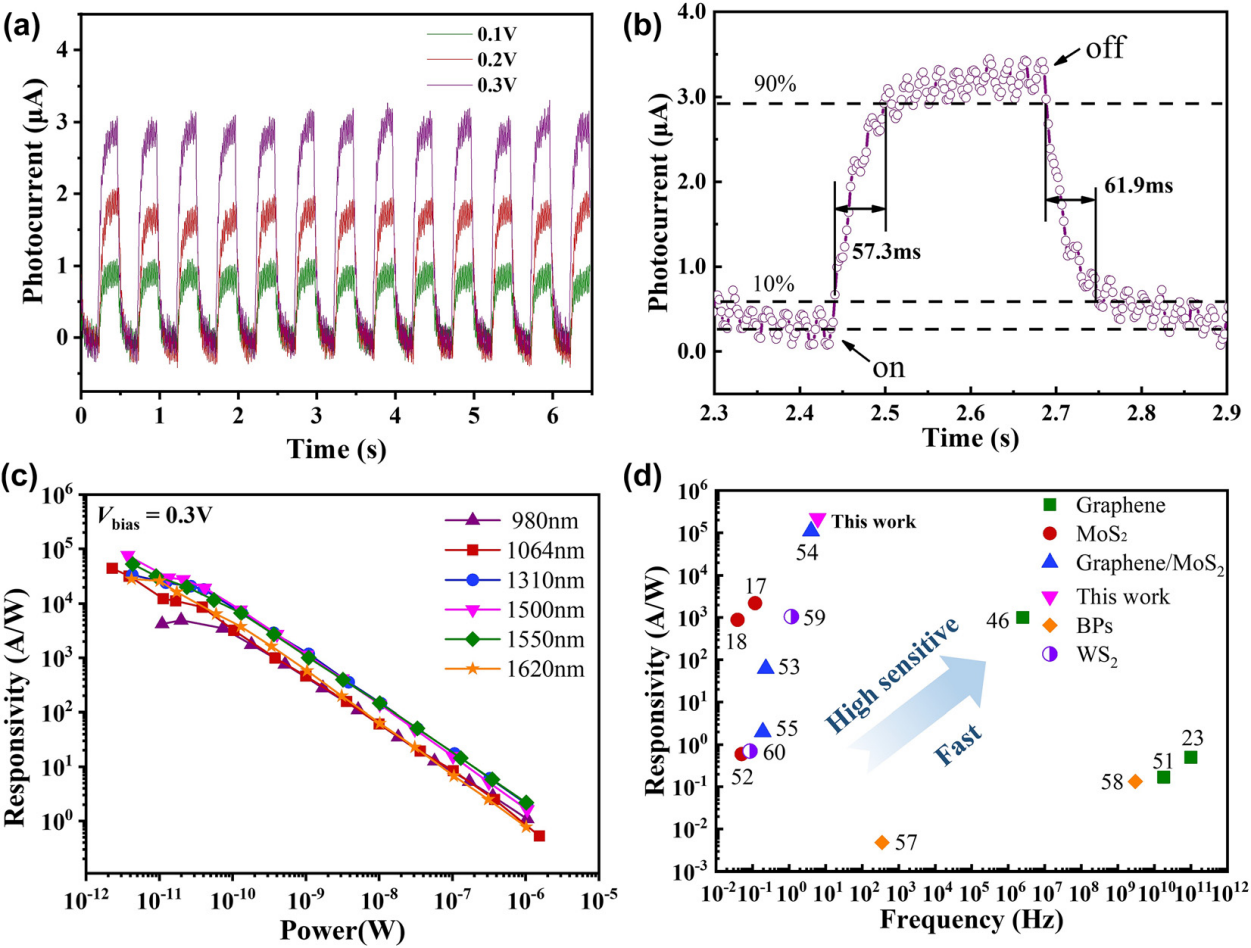
Time and wavelength dependent response. (a) Time dependent photocurrent of the device operating at a bias voltage of 0.1 V (black line), 0.2 V (red line), 0.3 V (blue line) at incident light power of 47.9 mW at 1550 nm; (b) the enlarged view of photocurrent at 0.3 V shows the rise time and the fall times are 57.3 and 61.9 ms, respectively; (c) responsivities as a function of the optical power for different illumination wavelengths (980, 1064, 1310, 1500, 1550 and 1620 nm); (d) comparison of the responsivity and bandwidth for the photodetectors based on graphene, MoS_2_, WS_2_, BPs and graphene/MoS_2_ heterostructures.

To further confirm the response time of the PPID, we measured the photocurrent temporal variation with a modulated laser under different bias voltages (0.1 V, 0.2 V, 0.3 V) at 1550 nm, as shown in Figure 6a. The incident light is modulated by a switching frequency of 2 Hz square pulse, and the photocurrent shows good repeatability and stability over 12-periods of light on–off. The photocurrents increase with the bias voltage, but the temporal responses are nearly identical. Figure 6b is the enlarge view of photocurrent under 0.3 V bias in Figure 6a, the rise and fall times are, respectively, 57.3 and 61.9 ms. The response time can be improved by improving the transfer process and shorten the spacing of the interdigitated Au finger electrode. Furthermore, we investigate the broadband detection ability of PPID for different incident wavelengths (980, 1064, 1310, 1500, 1550 and 1620 nm). The graphene/MoS_2_ heterostructures exhibit broadband wavelength dependence, graphene broadens the bandwidth of MoS_2_, and the PPID exhibit a strongest optical response at 1500 and 1550 nm. The photoresponsivity is larger than 4 kA W^−1^ at the incident light intensity of 10 pW at 0.3 V bias (in Figure 6c) in the wavelength range. In addition, Figure 6d shows the comparison of both the responsivity and bandwidth for the photodetectors based on graphene, MoS_2_, WS_2_, black phosphorus (BPs), and graphene/MoS_2_ heterostructures [17, 18, 23, 46, 51–55, 57–60]. The PPID exhibits a higher responsivity than graphene, MoS_2_, WS_2_ and BPs based photodetectors, and shows a relative faster response time than MoS_2_-based and WS_2_-based photodetectors. Although BPs-based photodetectors exhibit faster response time, but they suffer from instability in the air and low responsivity [53, 54]. The MoS_2_-based photodetectors suffer from long response time due to the low electron mobility (44 cm^2^ V^−1^ s^−1^) of MoS_2_, while graphene has higher electron mobility (>422.4 cm^2^ V^−1^ s^−1^), according to our previous work [20]. Constructing graphene/MoS_2_ heterostructures could increase the electron mobility of MoS_2_ and enhance the absorption of graphene, thus allowing to achieve a relative larger responsivity-bandwidth product for the photodetector.

**Figure 7:**
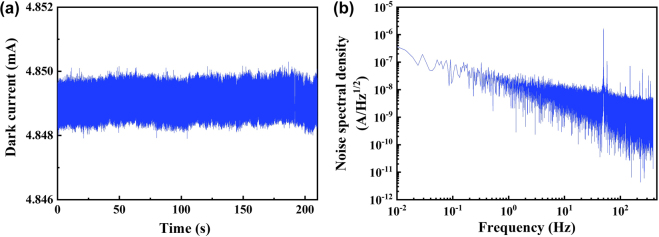
The NEP test results. (a) The dark current waveform of PPID at a bias voltage of 0.3 V and the sampling frequency of 1.95 KHz. (b) Analysis of noise spectral density of PPID based on the dark current waveform measured in (a).

Figure 7a shows the dark current at a bias voltage of 0.3 V, and the frequency dependent noise spectral density *S*_n_ is calculated by the dark current as shown in Figure 7b. The noise equivalent power (NEP) is defined as NEP = *S*_n_/*R* [54], it is found to be ∼9.5 × 10^−14^ W Hz^−1/2^ at 1 Hz, with *S*_n_ = ∼2.09 × 10^−8^ A Hz^−1/2^. The specific detectivity (D*) is defined as D* = *RA* ^1/2^/*S*_n_ [20], where *A* is the effective area of the photodetector, estimated as 8 μm × 3.5 mm = 2.8 × 10^−8^ m^2^. Thus, D* is calculated as 1.76 × 10^9^ Jones. The properties of small NEP and high responsivity indicate that the device has the potential application to detect ultra-weak photon emission from biological sample [60].

## 3 Conclusions

In summary, we integrated graphene/MoS_2_ heterostructures on the SPF to realize a novel high-performance multifunction-in-one optoelectronic device, which can simultaneously work as a polarizer and polarization-sensitive photodetector. In this work, Au film helps to enhance the TM mode absorption by 24.8 times, and graphene/MoS_2_ heterostructures help to enhance the TM mode absorption by 1.75 times and decrease the TE mode absorption by 2.2 times. Au film and graphene/MoS_2_ heterostructures enhanced the light absorption to improve the responsivity and contributed to the polarizing effect and polarization-sensitivity photodetection. Hence, the PPID realizes a high-responsivity (2.2 × 10^5^ A W^−1^) as a photodetector and a high PER (19.2 dB at *V*_bias_ = 4 V) as a polarizer. By optimizing the structure parameters, the PER and responsivity of the PPID could be further improved. This high-performance all-fiber device provides a feasible way for the detection of ultra-weak bioluminescence, and is also potential to be applied to polarization division (de) multiplexing and polarization optical fiber systems. Although it is still difficult to replace the commercial photodetectors, they are expected to be used in some special occasions where no need for fast response speed but require high responsivity. This kind of photodetectors are hopeful to become commercialized due to good stability, low cost, easy manufacturing, and high performance, but further research is still needed in integration and packaging technology.
